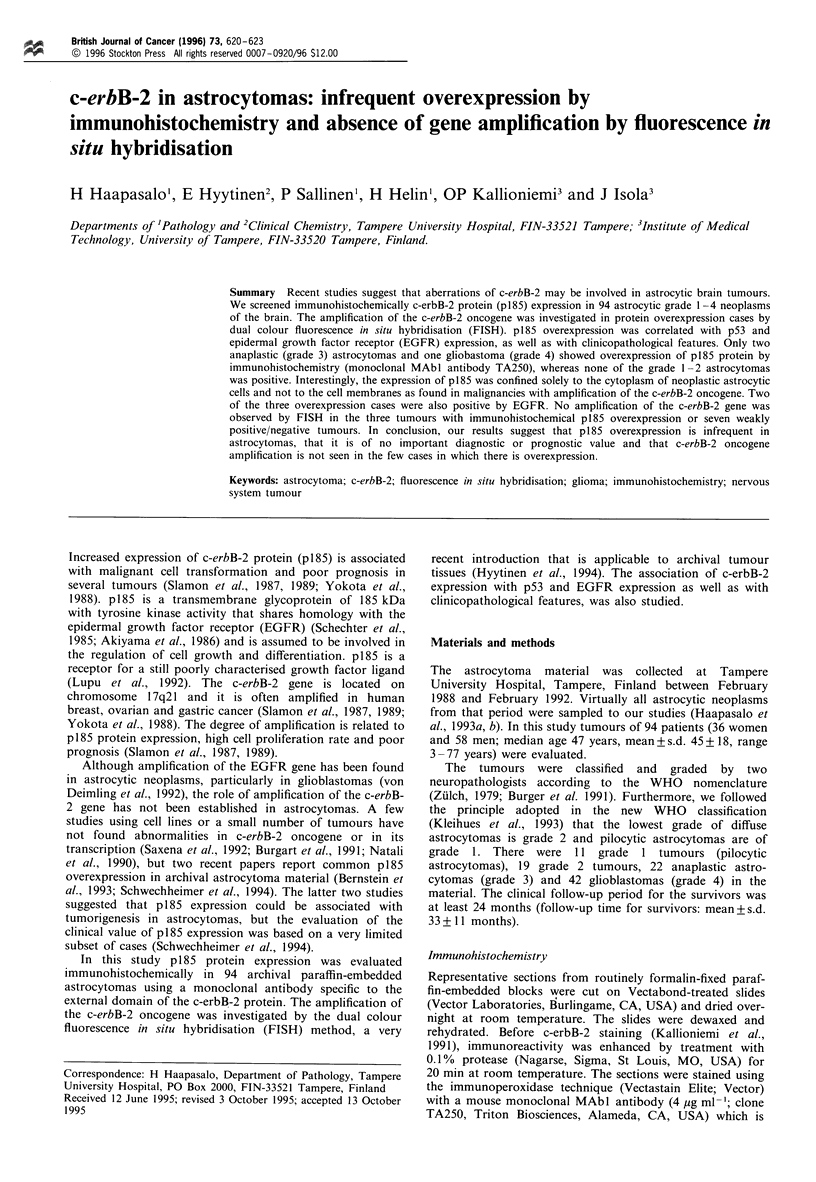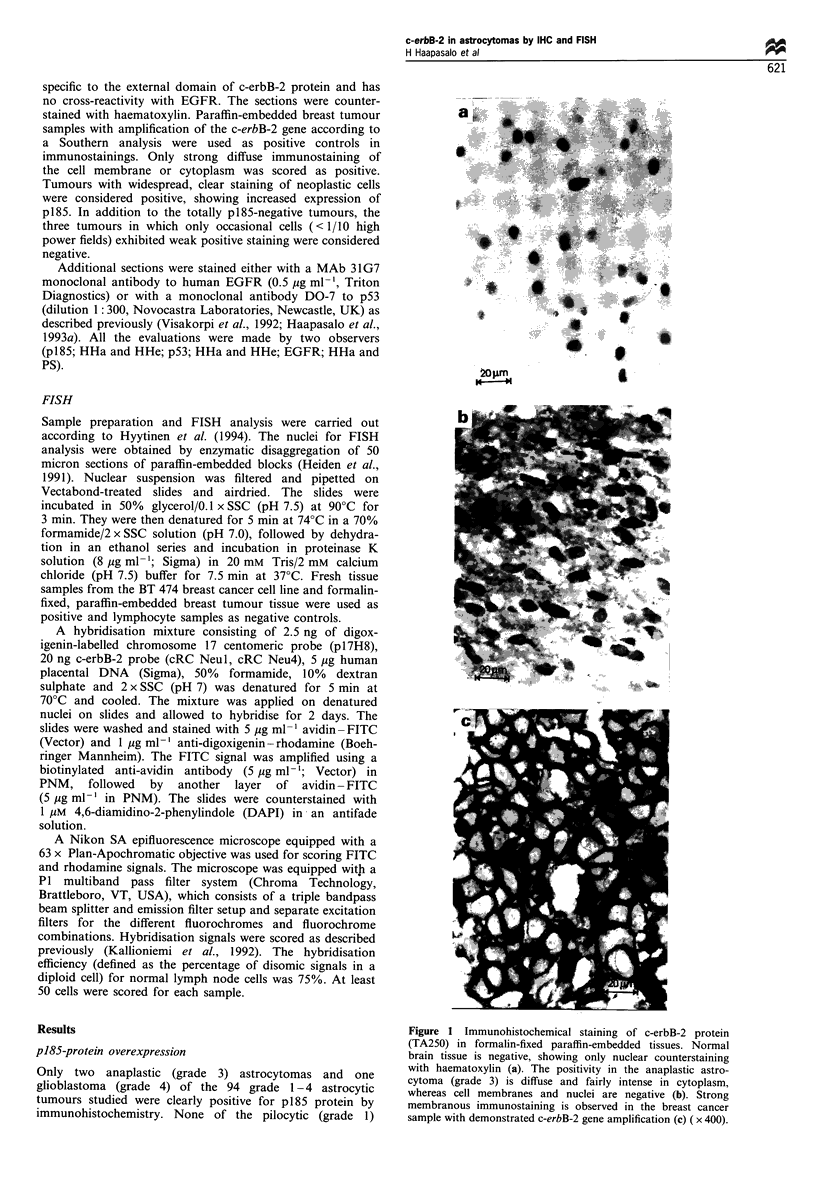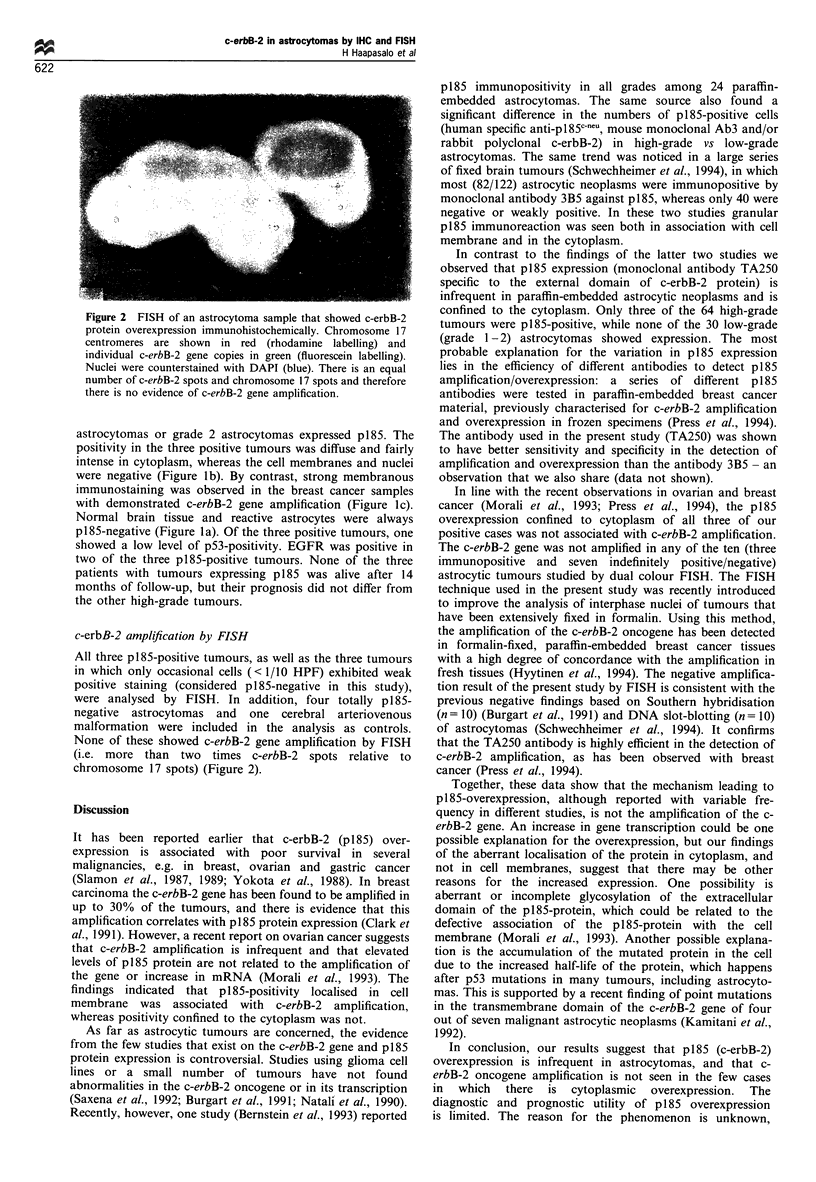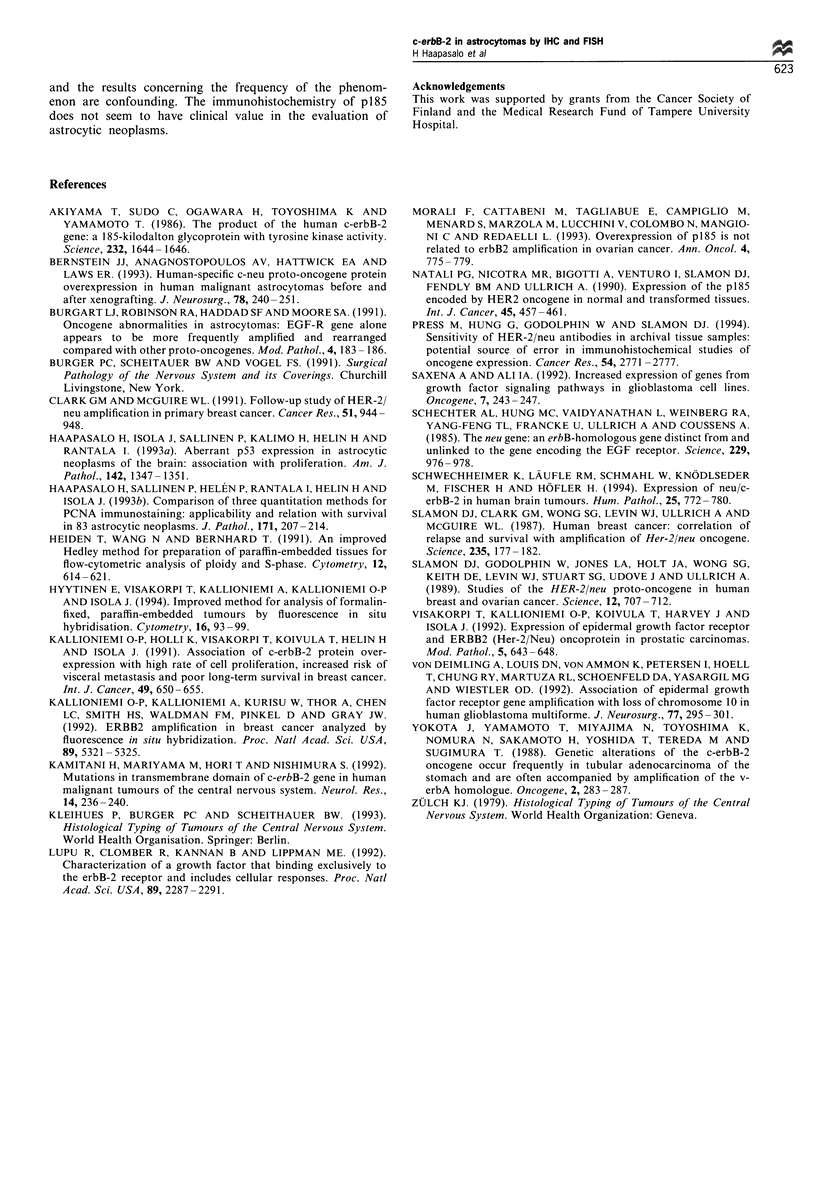# c-erbB-2 in astrocytomas: infrequent overexpression by immunohistochemistry and absence of gene amplification by fluorescence in situ hybridization.

**DOI:** 10.1038/bjc.1996.107

**Published:** 1996-03

**Authors:** H. Haapasalo, E. Hyytinen, P. Sallinen, H. Helin, O. P. Kallioniemi, J. Isola

**Affiliations:** Department of Pathology, Tampere University Hospital, Finland.

## Abstract

**Images:**


British Journal of Cancer (1996) 73, 620-623
© 1996 Stockton Press All rights reserved 0007-0920/96 $12.00

### c-*erb*B-2 in astrocytomas: infrequent overexpression by immunohistochemistry and absence of gene amplification by fluorescence *in situ* hybridisation

H Haapasalo[1], E Hyytinen[2], P Sallinen[1], H Helin[1], OP Kallioniemi[3] and J Isola[3]

*Departments of [1]Pathology and [2]Clinical Chemistry, Tampere University Hospital, FIN-33521 Tampere; [3]Institute of Medical Technology, University of Tampere, FIN-33520 Tampere, Finland.*

**Summary** Recent studies suggest that aberrations of c-*erb*B-2 may be involved in astrocytic brain tumours. We screened immunohistochemically c-erbB-2 protein (p185) expression in 94 astrocytic grade 1-4 neoplasms of the brain. The amplification of the c-*erb*B-2 oncogene was investigated in protein overexpression cases by dual colour fluorescence *in situ* hybridisation (FISH). p185 overexpression was correlated with p53 and epidermal growth factor receptor (EGFR) expression, as well as with clinicopathological features. Only two anaplastic (grade 3) astrocytomas and one gliobastoma (grade 4) showed overexpression of p185 protein by immunohistochemistry (monoclonal MAb1 antibody TA250), whereas none of the grade 1-2 astrocytomas was positive. Interestingly, the expression of p185 was confined solely to the cytoplasm of neoplastic astrocytic cells and not to the cell membranes as found in malignancies with amplification of the c-*erb*B-2 oncogene. Two of the three overexpression cases were also positive by EGFR. No amplification of the c-*erb*B-2 gene was observed by FISH in the three tumours with immunohistochemical p185 overexpression or seven weakly positive/negative tumours. In conclusion, our results suggest that p185 overexpression is infrequent in astrocytomas, that it is of no important diagnostic or prognostic value and that c-*erb*B-2 oncogene amplification is not seen in the few cases in which there is overexpression.

**Keywords:** astrocytoma; c-*erb*B-2; fluorescence *in situ* hybridisation; glioma; immunohistochemistry; nervous system tumour

Increased expression of c-*erb*B-2 protein (p185) is associated with malignant cell transformation and poor prognosis in several tumours (Slamon *et al.*, 1987, 1989; Yokota *et al.*, 1988). p185 is a transmembrane glycoprotein of 185 kDa with tyrosine kinase activity that shares homology with the epidermal growth factor receptor (EGFR) (Schechter *et al.*, 1985; Akiyama *et al.*, 1986) and is assumed to be involved in the regulation of cell growth and differentiation. p185 is a receptor for a still poorly characterised growth factor ligand (Lupu *et al.*, 1992). The c-*erb*B-2 gene is located on chromosome 17q21 and it is often amplified in human breast, ovarian and gastric cancer (Slamon *et al.*, 1987, 1989; Yokota *et al.*, 1988). The degree of amplification is related to p185 protein expression, high cell proliferation rate and poor prognosis (Slamon *et al.*, 1987, 1989).

Although amplification of the EGFR gene has been found in astrocytic neoplasms, particularly in glioblastomas (von Deimling *et al.*, 1992), the role of amplification of the c-*erb*B-2 gene has not been established in astrocytomas. A few studies using cell lines or a small number of tumours have not found abnormalities in c-*erb*B-2 oncogene or in its transcription (Saxena *et al.*, 1992; Burgart *et al.*, 1991; Natali *et al.*, 1990), but two recent papers report common p185 overexpression in archival astrocytoma material (Bernstein *et al.*, 1993; Schwechheimer *et al.*, 1994). The latter two studies suggested that p185 expression could be associated with tumorigenesis in astrocytomas, but the evaluation of the clinical value of p185 expression was based on a very limited subset of cases (Schwechheimer *et al.*, 1994).

In this study p185 protein expression was evaluated immunohistochemically in 94 archival paraffin-embedded astrocytomas using a monoclonal antibody specific to the external domain of the c-erbB-2 protein. The amplification of the c-*erb*B-2 oncogene was investigated by the dual colour fluorescence *in situ* hybridisation (FISH) method, a very recent introduction that is applicable to archival tumour tissues (Hyytinen *et al.*, 1994). The association of c-erbB-2 expression with p53 and EGFR expression as well as with clinicopathological features, was also studied.

### Materials and methods

The astrocytoma material was collected at Tampere University Hospital, Tampere, Finland between February 1988 and February 1992. Virtually all astrocytic neoplasms from that period were sampled to our studies (Haapasalo *et al.*, 1993*a*, *b*). In this study tumours of 94 patients (36 women and 58 men; median age 47 years, mean ± s.d. 45 ± 18, range 3-77 years) were evaluated.

The tumours were classified and graded by two neuropathologists according to the WHO nomenclature (Zülch, 1979; Burger *et al.* 1991). Furthermore, we followed the principle adopted in the new WHO classification (Kleihues *et al.*, 1993) that the lowest grade of diffuse astrocytomas is grade 2 and pilocytic astrocytomas are of grade 1. There were 11 grade 1 tumours (pilocytic astrocytomas), 19 grade 2 tumours, 22 anaplastic astrocytomas (grade 3) and 42 glioblastomas (grade 4) in the material. The clinical follow-up period for the survivors was at least 24 months (follow-up time for survivors: mean ± s.d. 33 ± 11 months).

### *Immunohistochemistry*

Representative sections from routinely formalin-fixed paraffin-embedded blocks were cut on Vectabond-treated slides (Vector Laboratories, Burlingame, CA, USA) and dried overnight at room temperature. The slides were dewaxed and rehydrated. Before c-erbB-2 staining (Kallioniemi *et al.*, 1991), immunoreactivity was enhanced by treatment with 0.1% protease (Nagarse, Sigma, St Louis, MO, USA) for 20 min at room temperature. The sections were stained using the immunoperoxidase technique (Vectastain Elite; Vector) with a mouse monoclonal MAb1 antibody (4 $\mu g\ ml^{-1}$; clone TA250, Triton Biosciences, Alameda, CA, USA) which is

Correspondence: H Haapasalo, Department of Pathology, Tampere University Hospital, PO Box 2000, FIN-33521 Tampere, Finland
Received 12 June 1995; revised 3 October 1995; accepted 13 October 1995

specific to the external domain of c-erbB-2 protein and has no cross-reactivity with EGFR. The sections were counterstained with haematoxylin. Paraffin-embedded breast tumour samples with amplification of the c-*erb*B-2 gene according to a Southern analysis were used as positive controls in immunostainings. Only strong diffuse immunostaining of the cell membrane or cytoplasm was scored as positive. Tumours with widespread, clear staining of neoplastic cells were considered positive, showing increased expression of p185. In addition to the totally p185-negative tumours, the three tumours in which only occasional cells (< 1/10 high power fields) exhibited weak positive staining were considered negative.

Additional sections were stained either with a MAb 31G7 monoclonal antibody to human EGFR (0.5 $\mu$g ml$^{-1}$, Triton Diagnostics) or with a monoclonal antibody DO-7 to p53 (dilution 1 : 300, Novocastra Laboratories, Newcastle, UK) as described previously (Visakorpi *et al.*, 1992; Haapasalo *et al.*, 1993*a*). All the evaluations were made by two observers (p185; HHa and HHe; p53; HHa and HHe; EGFR; HHa and PS).

### *FISH*

Sample preparation and FISH analysis were carried out according to Hyytinen *et al.* (1994). The nuclei for FISH analysis were obtained by enzymatic disaggregation of 50 micron sections of paraffin-embedded blocks (Heiden *et al.*, 1991). Nuclear suspension was filtered and pipetted on Vectabond-treated slides and airdried. The slides were incubated in 50% glycerol/0.1 × SSC (pH 7.5) at 90°C for 3 min. They were then denatured for 5 min at 74°C in a 70% formamide/2 × SSC solution (pH 7.0), followed by dehydration in an ethanol series and incubation in proteinase K solution (8 $\mu$g ml$^{-1}$; Sigma) in 20 mM Tris/2 mM calcium chloride (pH 7.5) buffer for 7.5 min at 37°C. Fresh tissue samples from the BT 474 breast cancer cell line and formalin-fixed, paraffin-embedded breast tumour tissue were used as positive and lymphocyte samples as negative controls.

A hybridisation mixture consisting of 2.5 ng of digoxigenin-labelled chromosome 17 centomeric probe (p17H8), 20 ng c-erbB-2 probe (cRC Neu1, cRC Neu4), 5 $\mu$g human placental DNA (Sigma), 50% formamide, 10% dextran sulphate and 2 × SSC (pH 7) was denatured for 5 min at 70°C and cooled. The mixture was applied on denatured nuclei on slides and allowed to hybridise for 2 days. The slides were washed and stained with 5 $\mu$g ml$^{-1}$ avidin–FITC (Vector) and 1 $\mu$g ml$^{-1}$ anti-digoxigenin–rhodamine (Boehringer Mannheim). The FITC signal was amplified using a biotinylated anti-avidin antibody (5 $\mu$g ml$^{-1}$; Vector) in PNM, followed by another layer of avidin–FITC (5 $\mu$g ml$^{-1}$ in PNM). The slides were counterstained with 1 $\mu$M 4,6-diamidino-2-phenylindole (DAPI) in an antifade solution.

A Nikon SA epifluorescence microscope equipped with a 63 × Plan-Apochromatic objective was used for scoring FITC and rhodamine signals. The microscope was equipped with a P1 multiband pass filter system (Chroma Technology, Brattleboro, VT, USA), which consists of a triple bandpass beam splitter and emission filter setup and separate excitation filters for the different fluorochromes and fluorochrome combinations. Hybridisation signals were scored as described previously (Kallioniemi *et al.*, 1992). The hybridisation efficiency (defined as the percentage of disomic signals in a diploid cell) for normal lymph node cells was 75%. At least 50 cells were scored for each sample.

### Results

### *p185-protein overexpression*

Only two anaplastic (grade 3) astrocytomas and one glioblastoma (grade 4) of the 94 grade 1–4 astrocytic tumours studied were clearly positive for p185 protein by immunohistochemistry. None of the pilocytic (grade 1)

**Figure 1** Immunohistochemical staining of c-erbB-2 protein (TA250) in formalin-fixed paraffin-embedded tissues. Normal brain tissue is negative, showing only nuclear counterstaining with haematoxylin (**a**). The positivity in the anaplastic astrocytoma (grade 3) is diffuse and fairly intense in cytoplasm, whereas cell membranes and nuclei are negative (**b**). Strong membranous immunostaining is observed in the breast cancer sample with demonstrated c-*erb*B-2 gene amplification (**c**) ( × 400).

**Figure 2** FISH of an astrocytoma sample that showed c-erbB-2 protein overexpression immunohistochemically. Chromosome 17 centromeres are shown in red (rhodamine labelling) and individual c-*erb*B-2 gene copies in green (fluorescein labelling). Nuclei were counterstained with DAPI (blue). There is an equal number of c-*erb*B-2 spots and chromosome 17 spots and therefore there is no evidence of c-*erb*B-2 gene amplification.

astrocytomas or grade 2 astrocytomas expressed p185. The positivity in the three positive tumours was diffuse and fairly intense in cytoplasm, whereas the cell membranes and nuclei were negative (Figure 1b). By contrast, strong membranous immunostaining was observed in the breast cancer samples with demonstrated c-*erb*B-2 gene amplification (Figure 1c). Normal brain tissue and reactive astrocytes were always p185-negative (Figure 1a). Of the three positive tumours, one showed a low level of p53-positivity. EGFR was positive in two of the three p185-positive tumours. None of the three patients with tumours expressing p185 was alive after 14 months of follow-up, but their prognosis did not differ from the other high-grade tumours.

*c-*erbB*-2 amplification by FISH*

All three p185-positive tumours, as well as the three tumours in which only occasional cells (< 1/10 HPF) exhibited weak positive staining (considered p185-negative in this study), were analysed by FISH. In addition, four totally p185-negative astrocytomas and one cerebral arteriovenous malformation were included in the analysis as controls. None of these showed c-*erb*B-2 gene amplification by FISH (i.e. more than two times c-*erb*B-2 spots relative to chromosome 17 spots) (Figure 2).

### Discussion

It has been reported earlier that c-erbB-2 (p185) overexpression is associated with poor survival in several malignancies, e.g. in breast, ovarian and gastric cancer (Slamon *et al.*, 1987, 1989; Yokota *et al.*, 1988). In breast carcinoma the c-*erb*B-2 gene has been found to be amplified in up to 30% of the tumours, and there is evidence that this amplification correlates with p185 protein expression (Clark *et al.*, 1991). However, a recent report on ovarian cancer suggests that c-*erb*B-2 amplification is infrequent and that elevated levels of p185 protein are not related to the amplification of the gene or increase in mRNA (Morali *et al.*, 1993). The findings indicated that p185-positivity localised in cell membrane was associated with c-*erb*B-2 amplification, whereas positivity confined to the cytoplasm was not.

As far as astrocytic tumours are concerned, the evidence from the few studies that exist on the c-*erb*B-2 gene and p185 protein expression is controversial. Studies using glioma cell lines or a small number of tumours have not found abnormalities in the c-*erb*B-2 oncogene or in its transcription (Saxena *et al.*, 1992; Burgart *et al.*, 1991; Natali *et al.*, 1990). Recently, however, one study (Bernstein *et al.*, 1993) reported p185 immunopositivity in all grades among 24 paraffin-embedded astrocytomas. The same source also found a significant difference in the numbers of p185-positive cells (human specific anti-p185$^{c\text{-}neu}$, mouse monoclonal Ab3 and/or rabbit polyclonal c-erbB-2) in high-grade *vs* low-grade astrocytomas. The same trend was noticed in a large series of fixed brain tumours (Schwechheimer *et al.*, 1994), in which most (82/122) astrocytic neoplasms were immunopositive by monoclonal antibody 3B5 against p185, whereas only 40 were negative or weakly positive. In these two studies granular p185 immunoreaction was seen both in association with cell membrane and in the cytoplasm.

In contrast to the findings of the latter two studies we observed that p185 expression (monoclonal antibody TA250 specific to the external domain of c-erbB-2 protein) is infrequent in paraffin-embedded astrocytic neoplasms and is confined to the cytoplasm. Only three of the 64 high-grade tumours were p185-positive, while none of the 30 low-grade (grade 1–2) astrocytomas showed expression. The most probable explanation for the variation in p185 expression lies in the efficiency of different antibodies to detect p185 amplification/overexpression: a series of different p185 antibodies were tested in paraffin-embedded breast cancer material, previously characterised for c-*erb*B-2 amplification and overexpression in frozen specimens (Press *et al.*, 1994). The antibody used in the present study (TA250) was shown to have better sensitivity and specificity in the detection of amplification and overexpression than the antibody 3B5 – an observation that we also share (data not shown).

In line with the recent observations in ovarian and breast cancer (Morali *et al.*, 1993; Press *et al.*, 1994), the p185 overexpression confined to cytoplasm of all three of our positive cases was not associated with c-*erb*B-2 amplification. The c-*erb*B-2 gene was not amplified in any of the ten (three immunopositive and seven indefinitely positive/negative) astrocytic tumours studied by dual colour FISH. The FISH technique used in the present study was recently introduced to improve the analysis of interphase nuclei of tumours that have been extensively fixed in formalin. Using this method, the amplification of the c-*erb*B-2 oncogene has been detected in formalin-fixed, paraffin-embedded breast cancer tissues with a high degree of concordance with the amplification in fresh tissues (Hyytinen *et al.*, 1994). The negative amplification result of the present study by FISH is consistent with the previous negative findings based on Southern hybridisation ($n = 10$) (Burgart *et al.*, 1991) and DNA slot-blotting ($n = 10$) of astrocytomas (Schwechheimer *et al.*, 1994). It confirms that the TA250 antibody is highly efficient in the detection of c-*erb*B-2 amplification, as has been observed with breast cancer (Press *et al.*, 1994).

Together, these data show that the mechanism leading to p185-overexpression, although reported with variable frequency in different studies, is not the amplification of the c-*erb*B-2 gene. An increase in gene transcription could be one possible explanation for the overexpression, but our findings of the aberrant localisation of the protein in cytoplasm, and not in cell membranes, suggest that there may be other reasons for the increased expression. One possibility is aberrant or incomplete glycosylation of the extracellular domain of the p185-protein, which could be related to the defective association of the p185-protein with the cell membrane (Morali *et al.*, 1993). Another possible explanation is the accumulation of the mutated protein in the cell due to the increased half-life of the protein, which happens after p53 mutations in many tumours, including astrocytomas. This is supported by a recent finding of point mutations in the transmembrane domain of the c-*erb*B-2 gene of four out of seven malignant astrocytic neoplasms (Kamitani *et al.*, 1992).

In conclusion, our results suggest that p185 (c-erbB-2) overexpression is infrequent in astrocytomas, and that c-*erb*B-2 oncogene amplification is not seen in the few cases in which there is cytoplasmic overexpression. The diagnostic and prognostic utility of p185 overexpression is limited. The reason for the phenomenon is unknown, and the results concerning the frequency of the phenomenon are confounding. The immunohistochemistry of p185 does not seem to have clinical value in the evaluation of astrocytic neoplasms.

**Acknowledgements**
This work was supported by grants from the Cancer Society of Finland and the Medical Research Fund of Tampere University Hospital.

### References

AKIYAMA T, SUDO C, OGAWARA H, TOYOSHIMA K AND YAMAMOTO T. (1986). The product of the human c-erbB-2 gene: a 185-kilodalton glycoprotein with tyrosine kinase activity. *Science*, **232,** 1644-1646.

BERNSTEIN JJ, ANAGNOSTOPOULOS AV, HATTWICK EA AND LAWS ER. (1993). Human-specific c-neu proto-oncogene protein overexpression in human malignant astrocytomas before and after xenografting. *J. Neurosurg.*, **78,** 240-251.

BURGART LJ, ROBINSON RA, HADDAD SF AND MOORE SA. (1991). Oncogene abnormalities in astrocytomas: EGF-R gene alone appears to be more frequently amplified and rearranged compared with other proto-oncogenes. *Mod. Pathol.*, **4,** 183-186.

BURGER PC, SCHEITAUER BW AND VOGEL FS. (1991). *Surgical Pathology of the Nervous System and its Coverings*. Churchill Livingstone, New York.

CLARK GM AND McGUIRE WL. (1991). Follow-up study of HER-2/neu amplification in primary breast cancer. *Cancer Res.*, **51,** 944-948.

HAAPASALO H, ISOLA J, SALLINEN P, KALIMO H, HELIN H AND RANTALA I. (1993*a*). Aberrant p53 expression in astrocytic neoplasms of the brain: association with proliferation. *Am. J. Pathol.*, **142,** 1347-1351.

HAAPASALO H, SALLINEN P, HELÉN P, RANTALA I, HELIN H AND ISOLA J. (1993*b*). Comparison of three quantitation methods for PCNA immunostaining: applicability and relation with survival in 83 astrocytic neoplasms. *J. Pathol.*, **171,** 207-214.

HEIDEN T, WANG N AND BERNHARD T. (1991). An improved Hedley method for preparation of paraffin-embedded tissues for flow-cytometric analysis of ploidy and S-phase. *Cytometry*, **12,** 614-621.

HYYTINEN E, VISAKORPI T, KALLIONIEMI A, KALLIONIEMI O-P AND ISOLA J. (1994). Improved method for analysis of formalin-fixed, paraffin-embedded tumours by fluorescence in situ hybridisation. *Cytometry*, **16,** 93-99.

KALLIONIEMI O-P, HOLLI K, VISAKORPI T, KOIVULA T, HELIN H AND ISOLA J. (1991). Association of c-erbB-2 protein over-expression with high rate of cell proliferation, increased risk of visceral metastasis and poor long-term survival in breast cancer. *Int. J. Cancer*, **49,** 650-655.

KALLIONIEMI O-P, KALLIONIEMI A, KURISU W, THOR A, CHEN LC, SMITH HS, WALDMAN FM, PINKEL D AND GRAY JW. (1992). ERBB2 amplification in breast cancer analyzed by fluorescence *in situ* hybridization. *Proc. Natl Acad. Sci. USA*, **89,** 5321-5325.

KAMITANI H, MARIYAMA M, HORI T AND NISHIMURA S. (1992). Mutations in transmembrane domain of c-*erb*B-2 gene in human malignant tumours of the central nervous system. *Neurol. Res.*, **14,** 236-240.

KLEIHUES P, BURGER PC AND SCHEITHAUER BW. (1993). *Histological Typing of Tumours of the Central Nervous System*. World Health Organisation. Springer: Berlin.

LUPU R, CLOMBER R, KANNAN B AND LIPPMAN ME. (1992). Characterization of a growth factor that binding exclusively to the erbB-2 receptor and includes cellular responses. *Proc. Natl Acad. Sci. USA*, **89,** 2287-2291.

MORALI F, CATTABENI M, TAGLIABUE E, CAMPIGLIO M, MENARD S, MARZOLA M, LUCCHINI V, COLOMBO N, MANGIONI C AND REDAELLI L. (1993). Overexpression of p185 is not related to erbB2 amplification in ovarian cancer. *Ann. Oncol.* **4,** 775-779.

NATALI PG, NICOTRA MR, BIGOTTI A, VENTURO I, SLAMON DJ, FENDLY BM AND ULLRICH A. (1990). Expression of the p185 encoded by HER2 oncogene in normal and transformed tissues. *Int. J. Cancer*, **45,** 457-461.

PRESS M, HUNG G, GODOLPHIN W AND SLAMON DJ. (1994). Sensitivity of HER-2/neu antibodies in archival tissue samples: potential source of error in immunohistochemical studies of oncogene expression. *Cancer Res.*, **54,** 2771-2777.

SAXENA A AND ALI IA. (1992). Increased expression of genes from growth factor signaling pathways in glioblastoma cell lines. *Oncogene*, **7,** 243-247.

SCHECHTER AL, HUNG MC, VAIDYANATHAN L, WEINBERG RA, YANG-FENG TL, FRANCKE U, ULLRICH A AND COUSSENS A. (1985). The *neu* gene: an *erb*B-homologous gene distinct from and unlinked to the gene encoding the EGF receptor. *Science*, **229,** 976-978.

SCHWECHHEIMER K, LÄUFLE RM, SCHMAHL W, KNÖDLSEDER M, FISCHER H AND HÖFLER H. (1994). Expression of neu/c-erbB-2 in human brain tumours. *Hum. Pathol.*, **25,** 772-780.

SLAMON DJ, CLARK GM, WONG SG, LEVIN WJ, ULLRICH A AND McGUIRE WL. (1987). Human breast cancer: correlation of relapse and survival with amplification of *Her-2/neu* oncogene. *Science*, **235,** 177-182.

SLAMON DJ, GODOLPHIN W, JONES LA, HOLT JA, WONG SG, KEITH DE, LEVIN WJ, STUART SG, UDOVE J AND ULLRICH A. (1989). Studies of the *HER-2/neu* proto-oncogene in human breast and ovarian cancer. *Science*, **12,** 707-712.

VISAKORPI T, KALLIONIEMI O-P, KOIVULA T, HARVEY J AND ISOLA J. (1992). Expression of epidermal growth factor receptor and ERBB2 (Her-2/Neu) oncoprotein in prostatic carcinomas. *Mod. Pathol.*, **5,** 643-648.

VON DEIMLING A, LOUIS DN, VON AMMON K, PETERSEN I, HOELL T, CHUNG RY, MARTUZA RL, SCHOENFELD DA, YASARGIL MG AND WIESTLER OD. (1992). Association of epidermal growth factor receptor gene amplification with loss of chromosome 10 in human glioblastoma multiforme. *J. Neurosurg.*, **77,** 295-301.

YOKOTA J, YAMAMOTO T, MIYAJIMA N, TOYOSHIMA K, NOMURA N, SAKAMOTO H, YOSHIDA T, TEREDA M AND SUGIMURA T. (1988). Genetic alterations of the c-erbB-2 oncogene occur frequently in tubular adenocarcinoma of the stomach and are often accompanied by amplification of the v-erbA homologue. *Oncogene*, **2,** 283-287.

ZÜLCH KJ. (1979). *Histological Typing of Tumours of the Central Nervous System*. World Health Organization: Geneva.